# Pain after uterine artery embolization with intrauterine device *in situ*

**DOI:** 10.1259/bjrcr.20190128

**Published:** 2020-03-20

**Authors:** Katherine Jane Chua, Bruce McLucas

**Affiliations:** 1 Department of Obstetrics & Gynecology, Saint Peter’s University Hospital, New Brunswick, NJ, USA; 2 Department of Obstetrics & Gynecology, University of California – Los Angeles, Los Angeles, CA, USA

## Abstract

Uterine artery embolization (UAE) is a minimally invasive option for females with symptomatic leiomyomas. Studies detailing a possible risk with an intrauterine device (IUD) *in situ* during UAE are limited. A 43-year-old female (Gravida 2, Para 2) underwent UAE with an IUD *in situ*. On post-procedure day 2, the patient presented with severe lower abdominal pain and mild leukocytosis. Following removal of her IUD, the patient experienced immediate pain relief. Caution is given to clinicians who wish to perform UAE with an IUD *in situ*.

## Clinical report

A 43-year-old female (Gravida 2, Para 2) presented with symptomatic leiomyoma with menorrhagia, dysmenorrhea and dyspareunia. She failed medical management with nonsteroidal anti-inflammatory drugs (NSAIDs) and hormone therapy. Pelvic ultrasound performed 3 months prior to her UAE, revealed multiple myomata, the largest measuring 5.6 cm in diameter and an IUD *in situ*. The patient had prior IUD (Mirena) insertions; one for 4.5 years, another lasting for 3.5 years and most recent IUD placed 14 months prior to UAE without complications.

UAE was performed under conscious sedation. The arterial circulation was entered through the right common femoral artery. Intravenous antibiotics (1g of cefazolin) was given for prophylaxis. Under fluoroscopy, a hunter 2 5 mm catheter (Merit) was inserted into the right uterine artery. One vial of 700–1000 micron polyvinyl alcohol (PVA) particles (Merit) was used to occlude the artery. Next, the left uterine artery was identified and occluded with one vial of 700–1000 micron PVA particles (Merit). Stasis was seen in both the right and left uterine arteries. The patient was discharged the same day in stable condition.

## Investigations

**Figure 1.**
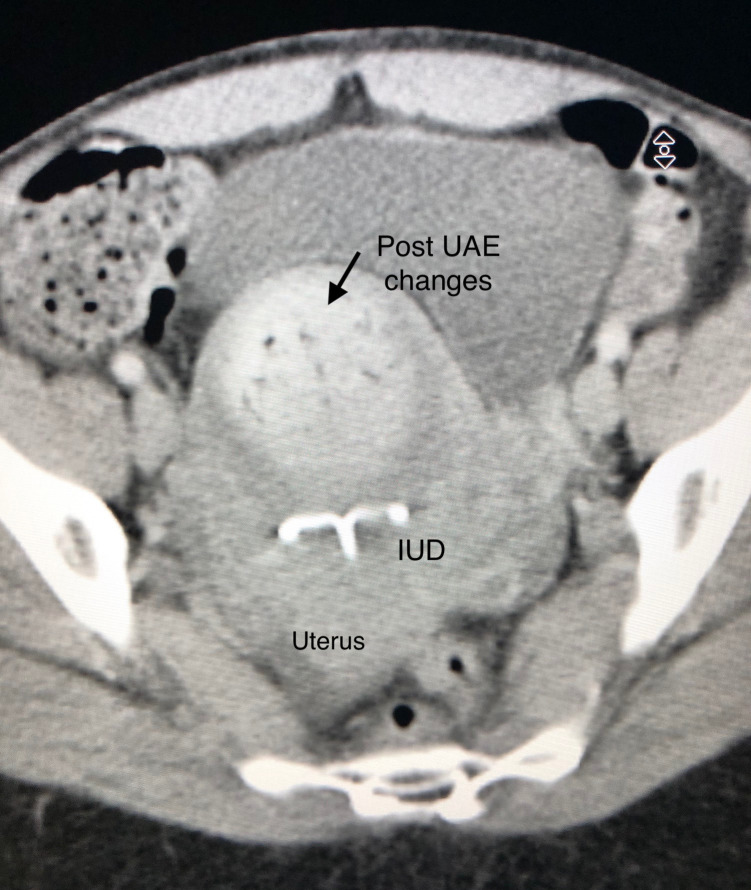
Siemens CT of the abdomen and pelvis with i.v. contrast in the portovenous phase demonstrates typical acute post-UAE changes within the fibroid. There is a small linear amount of gas and evidence of acute haemorrhagic infarction due to the high attenuation within the fibroids (arrow). The IUD is *in situ* and well within the endometrial cavity in proper position.

On post-procedure day 2, the patient developed severe lower abdominal pain and was admitted for further evaluation. She had received i.v. pain medication (i.v. ketorolac and i.v. hydromorphone) for pain control with limited relief. Siemens CT of the abdomen and pelvis with i.v. contrast revealed normal post-UAE changes within the fibroid. The high attenuation within the fibroid is likely due to acute haemorrhagic infarction. The IUD is in proper positioning within the uterine cavity with no evidence of abscess. (Figure 1). Her initial white blood cell count (WBC) was slightly elevated at 12.7 × 10^9^/L (normal range: 4.5–11.0 × 10^9^/L). She was empirically placed on piperacillin/tazobactam for a possible infection but remained afebrile throughout her hospital stay.

## Treatment

On post-procedure day 4 (hospital day 2), the patient’s pain persisted (9 out of 10). She was taken to the operating room for removal of her IUD. Pathology report of endometrial curetting revealed an acute endometritis. Blood, urine and endometrial cultures were negative.

## Outcome/Follow-up

Following removal of the IUD, the patient reported immediate pain relief. She was discharged in stable condition on post-procedure day 5 (hospital day 3). The patient is now 3-month post-UAE and has no further reports of pelvic pain with good symptomatic relief of menorrhagia and bloating.

## Discussion

Uterine artery embolization (UAE) is a minimal invasive option for females with symptomatic leiomyomas.^1^ Studies concerning the presence on an IUD during UAE are inconclusive. The theoretical risk of UAE with IUD *in situ* is due to an increased risk of infection post-procedure.^1–3^ A recent case series reviewing 20 patients has shown no evidence of infections following embolization with an IUD *in situ*.^3^

The IUD is one of the most widely used methods of reversible contraception.^1^ The IUD acts to create a sterile endometritis and prevents implantation of an embryo in the endometrial cavity.^1,4,5^ The pathology report confirms such an endometritis. The negative cultures ruled out an infection. The risk of pelvic inflammatory disease caused by an IUD is less than 1 in 1,300.^1,3^ Spies et al speculated that an IUD would increase the risk of post-procedure infection due the presence of a foreign body.^1^

Smeets et al recently published data with 20 patients who underwent UAE with the presence of an IUD. No IUD-related infectious processes took place up to 20-month follow-up. The authors suggested that an IUD in the presence of UAE should not be a relative contraindication.^1,3^

The patient had no evidence of an infectious aetiology for her pain. Typical air pockets within the myometrium have been described in numerous series and indeed are seen in our patient.^6^ There is no evidence of a pelvic abscess. Furthermore, the immediate relief of the patient’s pain following the removal of the IUD is notable. Based on this experience, caution is recommended to clinicians when performing UAE with IUD *in situ*. Larger studies are indicated.

## Learning points

- No large-scale studies have been conducted regarding UAE with IUD *in situ*. There is a theoretical risk of increased infection rates associated with performing UAE with IUD *in situ*.
- Although, a recent case series of 20 patients revealed no evidence of infection following embolization with UAE with IUD *in situ*, the patient presented experienced immediate pain relief following removal of IUD a few days after UAE was performed *in situ*. Further investigation is required.
- Caution is advised to clinicians when performing UAE with IUD *in situ*.
